# Study on the Characteristics of Fine Rice Flour by Micro-Crushing and Its Effects on the Quality Improvement of Rice Cakes

**DOI:** 10.3390/foods13223565

**Published:** 2024-11-07

**Authors:** Mingshou Lu, Wanshan Yang, Huining Zhang, Yang Yu, Fenglian Chen, Yanling Hao

**Affiliations:** 1 College of Food Engineering, Harbin University of Commerce, Harbin 150028, China; lumingshou@hrbcu.edu.cn (M.L.); m13555016561@163.com (W.Y.); lemonzhn0923@163.com (H.Z.); yyhsd2024@163.com (Y.Y.); 2 Department of Nutrition and Health, China Agricultural University, Beijing 100091, China

**Keywords:** fine rice powder, mesh size, rice cake, quality improvement, sensory evaluation

## Abstract

To investigate the impact of micro-crushing technology on rice flour characteristics and its enhancement of rice cake quality, resting angle, slip angle, solubility, water-holding capacity, emulsification, oil absorption, gelatinization, and baking quality, rice flour and rice cakes were analyzed using a texture analyzer and a gelatinization instrument. The results indicated that a decrease in particle size led to a significant increase in damaged starch from 18% to 26.5%. Both the resting angle and slip angle increased, indicating reduced fluidity. The gelatinization temperature decreased from 76.63 °C to 71.61 °C, the gelatinization time was reduced from 6.6 min to 6.4 min, and gelatinization viscosity initially increased and then decreased. Water-holding capacity increased 1.0-fold with decreased particle size, and solubility increased 11.6-fold together with the increase temperature. Emulsification and oil absorption were optimal when the particle size exceeded 120–140 mesh. The conductivity of the rice flour suspension rose with decreased particle size, while the conductivity of rice flour paste and cake batter decreased. Decreased particle size led to significantly reduced rice cake hardness and chewiness of 47.77% and 52.44%, while elasticity, restoration, specific volume, and porosity increased 18.75%, 15.15%, 31.16% and 25.10%, respectively. The skin and core color darkened with reduced luminosity, correlating with enhanced sensory scores. Correlation analysis revealed that physiochemical properties of rice flour influenced gelatinization properties, thereby affecting rice cake quality. This study provides a foundation for utilizing fine rice flour in rice cakes.

## 1. Introduction

Traditional cakes, primarily made from wheat flour, contain wheat gluten (including gliadin and glutenin) that can trigger an immune response in individuals with celiac disease. The ingestion of these proteins can lead to inflammation of the villi in the small intestine, disrupting nutrient absorption. The sole effective treatment for celiac disease is a lifelong gluten-free diet, which gradually restores the intestinal mucosa [1]. Rice, a staple food for over half of the world’s population, features hypoallergenic rice protein, making it a common ingredient in food for infants, the elderly, and individuals allergic to gluten protein [2]. Additionally, rice processing generates broken rice by-products, which are economically priced [3]. Using rice flour to make rice cakes not only satisfies the demand for dietary diversity but also offers a way to effectively utilize broken rice resources [4].

Rice flour has been used as a substitute for wheat flour to enhance the rice cake-making process. Liu et al. [5] found that adding 5% glutinous rice flour of total ingredients resulted optimal cake quality compared to traditional wheat flour cake. Kim et al. [6] presented that when using japonica rice flour as a raw material, the quality of rice cake was improved with a soft texture, which provided a theoretical foundation for industrial rice cake production. Additionally, Lin et al. [7] found that rice cakes maintained a soft texture and delayed starch setback during storage, thus effectively extending the cake’s shelf life. In recent years, micro-crushing technology has gained considerable attention in the food raw materials field [8]. Fine powder, typically with a particle size of less than 100 μm, or defined as material that passes through a 150-mesh sieve (with a pore size of 90 μm ± 4.6 μm) and has a pass rate of not less than 95% through a 200-mesh sieve (with a pore size of 75 μm ± 4.1 μm), has been increasingly studied [9]. Micro-crushing is a processing method that applies high shear force and strong impact to materials, resulting in uniform and dense properties of raw material. It disrupts the material’s structural form without altering its molecular structure, enhancing its physicochemical and sensory properties and increasing the content of active substances to a certain extent. Meanwhile, Shi et al. [10] discovered that micro-crushing significantly enhanced the physical properties of wheat bran dietary fiber, leading to a 4.0-fold increase in water-holding capacity (WHC) and a 1.9-fold increase in oil-holding capacity compared to conventional crushing method. However, over-crushing damages the material structure, leading to the loss of hydrogen bonds and van der Waals forces within the material, which in turn affects its functional activity. In contrast, materials produced through micro-crushing exhibit various physicochemical and bioactive characteristics, such as high flowability and adsorptivity. Consequently, it is an efficient and environmentally friendly physical processing technology.

Therefore, this study addressed the practical requirements of industrial production by utilizing broken rice as a raw material. The rice was micro-crushed to 100 to 180 meshes and applied in rice cake production. Texture analysis and sensory evaluation of the cake were conducted, laying a solid foundation for enhancing the quality and processing of baked products using fine rice flour as a raw material.

## 2. Materials and Methods

### 2.1. Materials and Reagents

Rice flour was purchased from Xinglongwang Co., Ltd., Wuchang City, Heilongjiang Province, China. Soybean oil was purchased from Shandong Luhua Group Co., Ltd. (Laiyang, China). Petroleum ether and HCL were purchased from Tianjin Damao Chemical Reagent Factory (Tianjin, China), ethanol was purchased from Tianjin Zhiyuan Chemical Reagent Co., Ltd. (Tianjin, China), methylene blue was purchased from Tianjin Opsheng Chemical Co., Ltd. (Tianjin, China), methyl red was purchased from Tianjin Fengchuan Chemical Reagent Technology Co., Ltd. (Tianjin, China), copper sulfate was purchased from Tianjin Tianda Chemical Reagent Factory (Tianjin, China) and sodium hydroxide was purchased from Tianjin KaiTong Chemical Reagent Co., Ltd. (Tianjin, China). All chemicals were analytically pure reagents.

### 2.2. Fine Rice Flour Preparation

The rice flour was coarsely ground and then further crushed by an electrical pulverizer (2500Y; Xulang Machinery Equipment; Guangzhou, China). The resulting rice flour was then sieved by different meshes to obtain different particle sizes: 100 mesh (150 μm)–120 mesh (125 μm), 120 mesh–140 mesh (106 μm), 140 mesh–160 mesh (95 μm), and 160 mesh–180 mesh (90 μm). A greater mesh size represents a smaller grain size.

### 2.3. Fine Rice Flour Characterization

#### 2.3.1. Basic Components

Moisture content of rice flour was determined using the direct drying method, following reference GB 5009.3-2016 [11]. Protein content was determined using an automatic Kjeldahl apparatus (K9840; Haineng Scientific Instrument; Shanghai, China), following reference GB 5009.5-2016 [12]. The damaged starch content was determined using a damaged starch meter (SD-MATIC; Chopin Technologies; Villeneuve-la-Garenne, France), and starch content was determined using the acid hydrolysis method, following reference GB 5009.9-2023 [13]. A laser particle size analyzer (Nano-ZS90; Malvern Panalytical; Malvern, UK) was utilized to measure the average particle size of the fine rice flour, expressed as D50 [14]. The rice starch granule samples were observed under a 200× microscope (WYS-41XD; VIYEE Optics; Tianjin, China).

#### 2.3.2. Rest Angle and Slip Angle

Rice flour of varying mesh sizes was allowed to flow vertically through a glass funnel onto a glass plate. The distance from the tip of the funnel to the plate was 3.0 cm (H), and as the flour flowed down, it formed a cone on the plate with a diameter of 2R. The angle between the cone’s surface and the water level was measured as the rest angle for different particle size ranges of rice flour, calculated as Rest angle θ = arctg (2R/H) [15]. Rice flour of 5 g with different mesh sizes was placed on a 13 cm-long glass plate. The plate was then tilted until the flour began to move, and the angle between the plate and the water level was measured as the slip angle for different particle size ranges of rice flour, calculated as Slip angle α = arcsin (H/L) [16].

#### 2.3.3. Solubility and WHC

A precise amount of dry sample M (g) was weighed and adjusted to a (dry basis) concentration of 2% rice flour milk. The mixture was stirred at temperatures of 60 °C, 80 °C, and 100 °C for 30 min, followed by centrifugation at 3000 r/min for 20 min to separate the supernatant and precipitate. The supernatant was then evaporated in a water bath and dried in an oven at 105 °C to a constant weight. The mass of dissolved rice flour M1 (g) was obtained by weighing, and solubility (S) was calculated as S(%) = (M_1_/M) × 100 [17].

Next, 1.0 g of the sample (M_0_) was weighed and placed in a pre-weighed centrifuge tube. Water was gradually added while stirring the sample until the sample formed a slurry and anhydrous precipitates (M_1_). The mixture was centrifuged at 2000 r/min for 10 min and the total mass of the precipitate with centrifuge tube was M_2_. Then, WHC was calculated as WHC (g) = (M_2_ − M_1_)/M_0_ [18].

#### 2.3.4. Emulsifying Property and Oil-Absorbing Property

The sample was weighed at 0.5 g and was dissolved in 10 mL of distilled water. An equal volume of soybean oil was added, and the mixture was stirred with a magnetic stirrer at 1800 r/min for 10 min to ensure full dissolution. The liquid was then transferred to a centrifuge tube, and the height of the liquid level was noted (H_1_). After centrifugation at 1500 r/min for 10 min, the height of the upper emulsification layer was measured (H_2_), and the emulsifying property was calculated as emulsifying property (%) = (H_2_/H_1_) × 100 [19].

The sample was weighed at 0.5 g (M_0_) and was added to a centrifuge tube (total M_1_). Soybean oil of 5 mL was added to the tube, mixed for 1 min, and the mixed sample was dispersed in oil for 30 min, followed by centrifugation at 2000 r/min for 25min; the free oil was removed, and the remaining mass of precipitate and tube was M_2_ and the oil-absorbing property was calculated as oil-absorbing property (g/g) = (M_2_ − M_1_)/M_0_ [20].

#### 2.3.5. Electrical Conductivity and Pasting Properties

The electrical conductivity was measured using a conductivity tester (DDS-307; INESA Scientific Instrument, Shanghai, China). A suspension was prepared by mixing 1 g of rice flour with 10 mL of water. Then, 1 mL of NaCl (0.01 mol/L) was added as an electrolyte, and the conductivity of rice flour suspension was measured at 20 °C. A paste was made by mixing 1 g of rice flour with 10 mL of water and oscillating it evenly in a water bath at 90 °C for 10 min. Subsequently, 1 mL of NaCl (0.01 mol/L) was added as an electrolyte, and the conductivity of rice flour paste was determined at 20 °C. Cake batter was prepared using rice flour according to the production steps of clear cake [20]. Then, 1 mL of NaCl (0.01 mol/L) was added as an electrolyte to the cake batter, and the conductivity of the cake batter was measured at 20 °C.

Rapid viscometer analysis (RVA, TECMASTER Fast viscometer; Botong Technologies, Stockholm, Sweden) was utilized to determine the pasting properties of the sample [21]. The rice flour sample was accurately weighed at 3 g on a dry basis, then added with 25 g of distilled water. The detecting parameters were set as follows: the rotation speed was reduced from 960 r/min to 160 r/min within 10 s and maintained to 50 °C for 1 min. Next, the temperature was increased to 95 °C at a rate of 12 °C/min and maintained for 2.5 min, followed by cooling to 50 °C at a rate of 12 °C/min and holding for 1 min.

### 2.4. Rice Cake Preparation

For the basic recipe of rice cake, the quantity of rice flour was taken as the benchmark (100%); whole egg liquid, sugar, water, and baking powder accounted for 80%, 80%, 20%, and 1%, respectively [20]. All the ingredients were accurately weighed. The whole egg liquid was mixed with sugar and stirred with a whisk, simultaneously adding water twice until the bubbles of the egg liquid became fine and stable. Then rice flour and baking powder were added to the beaten whole egg liquid and mixed to obtain a rice flour batter. The batter samples were transferred to the cake mold, taking about 65% of the volume of the mold, and immediately placed into the oven. The cake was baked under 150 °C on top and 160 °C on bottom for 15 min.

### 2.5. Rice Cake Characterization

#### 2.5.1. Texture Analysis and Specific Volume

The rice flour cake was cooled and stabilized at room temperature for 20 min after baking, and a texture analyzer (TAnew plus; Ruifen Instrument; Shanghai, China) was used to perform texture profile analysis (TPA). Samples were taken from the uniformly dense center of the rice flour cake and cut into small pieces measuring 2 cm × 2 cm × 2 cm. The testing was conducted using a flat-bottomed cylindrical probe P/35 with a diameter of 35 mm. The testing parameters were adjusted as follows. Test type: compression; target mode: deformation; deformation amount: 50%; test speed: pre-test speed of 2.0 mm/s, test speed of 1.0 mm/s, post-test speed of 1.0 mm/s; contact type: force; contact point: 5.

The prepared rice cake was accurately weighed for its mass using a balance and then the millet substitution method was used to measure the cake’s volume. The specific volume (mL/g) of the cake was calculated as the division of the cake’s volume and the cake’s mass. The rice cake core was selected to take photos, and porosity analysis was performed using Image J 1.51j8 software.

#### 2.5.2. Color Analysis and Sensory Assessment

Color analysis of the outer skin and core of the cakes was conducted with a colorimeter (CM23D; Konica Minolta; Tokyo, Japan), and the L*, a*, b* and h values were recorded. The flour rice cake was left and demolded at room temperature, and ten volunteers were invited to conduct sensory evaluations by selecting appropriate sensory evaluation criteria based on shape, color, texture, flavor, and impurities. The evaluation criteria are shown in Table 1, with a maximum score of 100 points [22].

### 2.6. Statistical Analysis

All data were exhibited as mean ± standard deviation and plotted by Origin 2018. The analysis of variance and correlation of the data was performed using SPSS 17.0 software, the Duncan test was selected for ANOVA, and the data were statistically analyzed at the *p* < 0.05 test level. All experiments were carried out in triplicate.

## 3. Results and Discussion

### 3.1. Effect of Mesh Size on Properties of Fine Rice Flour

#### 3.1.1. Basic Components of Fine Rice Flour

Table 2 presents the basic components of rice flour granules with varying mesh sizes. It was evident from the table that the water content decreased as the mesh size increased. This decline was likely due to water loss from heat generated during the rice flour grinding process. Protein and total starch contents remained relatively stable across different mesh sizes. However, the content of damaged starch notably increased, particularly in the 160–180 mesh range, where it reached 26.05%. This was attributed to heat or mechanical force damage during the grinding process, which damaged the outer cell membrane of intact starch granules, leading to an increase in damaged starch. Smaller grain sizes of rice flour resulted in more thorough crushing, higher degrees of starch granule destruction, and increased damaged starch contents [23].

#### 3.1.2. The Flowability of Fine Rice Flour

**Figure 1:**
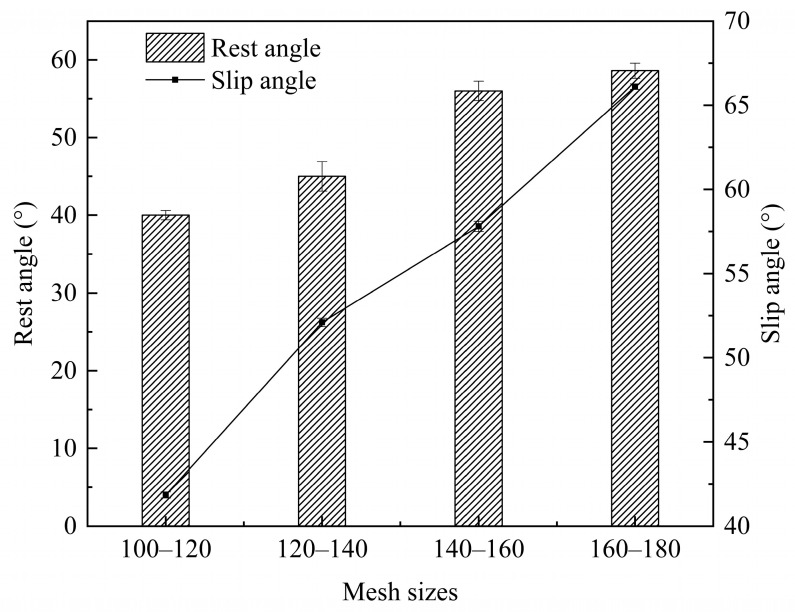
Rest angle and slip angle of rice flour sieved through different mesh sizes.

The rest angle and slip angle are indicative of powder flow performance and frictional properties. A smaller rest angle suggests better powder fluidity, while a larger angle indicates the opposite. A decrease in particle size (larger mesh size) led to a significant increase in both rest and slip angles (Figure 1), the rest angle increased from 40° to 58.6° and the slip angle increased from 41.85° to 66.1°. This considerable increase suggested that the powder exhibited strong adsorption and cohesion. This could be attributed to the fact that smaller rice flour particles had a larger specific surface area, resulting in enhanced electrostatic and surface polymerization forces. As a result, these particles were more prone to adsorption and agglomeration, leading to poorer fluidity [24].

#### 3.1.3. Solubility and Water Holding Capacity (WHC) of Fine Rice Flour

**Figure 2:**
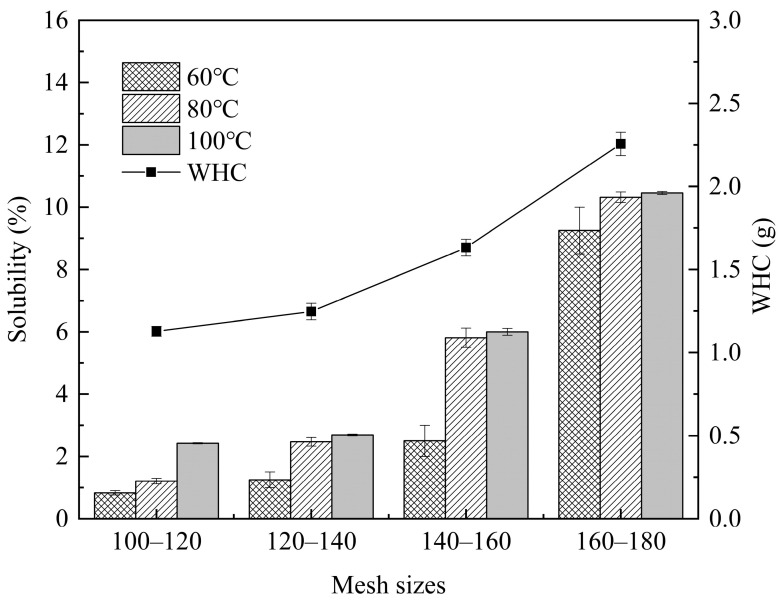
Solubility and WHC of rice flour sieved through different mesh sizes.

The solubility of starch reflects the internal bond strength within particles [25]. A decrease in particle size and an increase in temperature tended to elevate the solubility of rice flour (Figure 2). At the same temperature, the particle size peaked at 160 to 180 mesh. The increasing solubility of rice flour with decreasing particle size indicated that smaller particles exhibited enhanced dissolution performance and stronger dispersion. While rice flour was initially resistant to dissolving in water at room temperature, higher temperatures disrupted the hydrogen bonds in the starch molecule’s crystalline region, causing cracks in the crystal structure’s surface. This allowed free water to penetrate the starch molecule more easily, leading to increased solubility with rising temperatures [26].

Furthermore, the WHC of rice flour also increased with decreasing particle size. The mechanical forces involved in the micro-crushing process significantly altered the morphology of starch particles in rice, breaking them into numerous smaller particles. This process increased surface energy, specific surface area, porosity, and active points. Simultaneously, micro-crushing destroyed the lattice structure of starch and dissociated its double-helix structure. These mechanical effects greatly enhanced the combination of free hydroxyl groups of water molecules with starch molecules [27]. Consequently, smaller particle sizes of rice flour exhibited increased solubility and WHC.

#### 3.1.4. Emulsifying and Oil-Absorbing Properties of Fine Rice Flour

**Figure 3:**
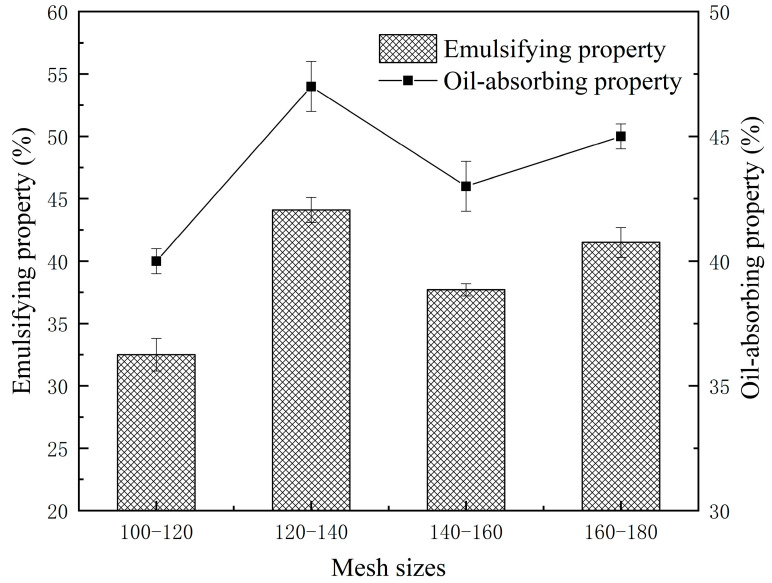
Emulsifying and oil-absorbing properties of rice flour sieved through different mesh sizes.

In this study, we investigated the cake batter formed prior to rice cake production as a heterogeneous dispersion system. This system includes gas, liquid, and solid components, as well as an oil–water dispersed emulsion. To explore the impact of emulsion balance on the final cake product, the emulsification and oil-absorbing properties of rice flour were analyzed. Emulsifying property refers to the ability of proteins in rice flour to act as surfactants at the oil–water interface [28]. It is evident that as particle size decreased, emulsifying and oil-absorbing properties increased, reaching optimal levels at 120–140 mesh, with values of 41.7% and 43%, respectively (Figure 3). In contrast, the values of 100–120 mesh rice flour were relatively poor, at only 32.5% and 40%, respectively. This trend was attributed to the breakdown of rice flour particles by mechanical forces, leading to reduced particle size, protein degradation, and increased hydrophobic groups. These changes facilitated protein interaction with non-polar solvents, enhancing its adsorption at the water–oil interface and improving the emulsification and oil absorption properties of rice flour [29].

#### 3.1.5. The Conductivity of Fine Rice Flour

Conductors can be categorized based on their method of conducting electricity. Metals fall into the first category, conducting electricity through the migration of free electrons. The second category comprises electrolyte solutions, which conduct electricity through the migration of ions. Most food materials belong to the second category, typically existing as solids, gels, or liquids [30]. Ions are the primary carriers of conduction current in a solution. Therefore, the conductivity of a solution is generally proportional to the number of ions present. A higher concentration of ions per unit volume of solution results in greater conductivity.

For rice flour, the conductivity increased slightly with the increase in mesh size but did not change much (Table 3). With the increase in water volume fraction, the conductivity gradually decreased; the change was most obvious when the water volume increased from 20% to 40%, which was due to the increase in water volume in the swelling process, the volume expansion of starch particles led to a decrease in the distance between them, the amount of unbound water decreased, resulting in a decrease in the area of charged particle movement and in the fluidity of water molecules, leading to a decrease in conductivity [31].

The conductivity of the gelatinized rice flour paste decreased with increasing water volume fraction, in contrast, indicating a reduction in ions caused by rice flour gelatinization. Upon reaching the gelatinization temperature, the swelling and gelatinization of starch in the rice flour led to new starch–water interactions, reducing free water and increasing bound water. This, coupled with the increase in combined water, resulted in a decrease in conductivity with increasing water volume fraction [32]. There are two mechanisms behind the decrease in conductivity after gelatinization. One is the decrease in ion migration rate as the gelatinization temperature decreases, while the other is the combination of ions and polar molecules due to the gelation process [33]. Starch in rice flour converted free water to bound water during gelatinization, a process requiring energy. Therefore, conductivity and ion migration rate were directly related to the charge carrier mechanism depending on system energy [34]. As the particle size decreased, gelatinization temperature decreased, gelatinization time shortened, and gel formation occurred more rapidly, resulting in fewer ions in the solution and, hence, lower conductivity.

It is evident that the conductivity of the cake batter decreased with higher water volume fractions. Notably, when the moisture volume fraction ranged from 80% to 100%, the conductivity of the cake batter was at its lowest. The most significant decrease in conductivity was observed as the moisture volume fraction increased from 20% to 40%. This trend might be attributed to the higher dilution of ions in batter prepared with higher water content in cake batter systems containing eggs and sugar, resulting in a lower total conductivity [35]. Also, the smaller particle size of rice flour leads to increased viscosity of the starch suspension, reduction of unbound water, and decreased mobility and conductivity of ions.

#### 3.1.6. Gelatinizing Properties of Fine Rice Flour

**Figure 4:**
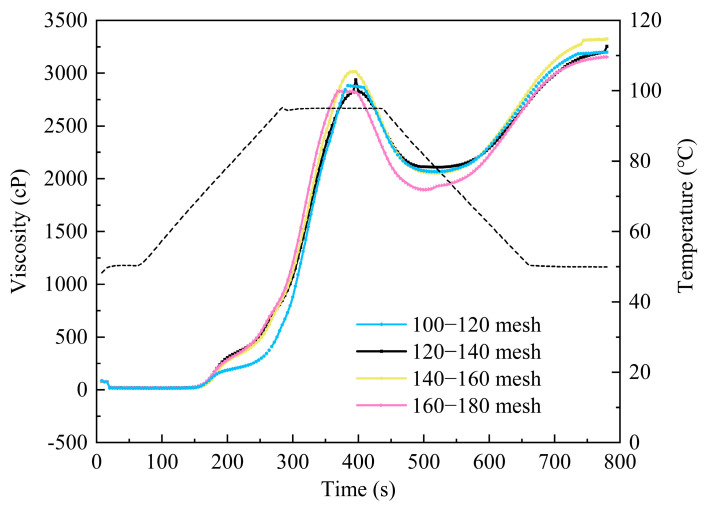
RVA characteristics of rice flour sieved through different mesh sizes.

During the baking process, rice flour undergoes glass transition, particle expansion, and gelatinization. Gelatinization, in essence, involves water penetrating the crystalline regions, disrupting the internal structure of starch particles. Consequently, the expansion rate and gelatinization viscosity are crucial factors influencing rice cake expansion [36]. RVA curves and primary gelatinization factors were observed to display the gelatinizing properties of fine rice flour (Figure 4 and Table 4). It is evident that as the particle size of rice flour decreased, the gelatinization temperature and time exhibited a downward trend. Peak viscosity and final viscosity initially increased and then decreased, while setback and breakdown values showed an increasing trend. Heating the rice flour solution weakened the hydrogen bonds between starch granules, leading to water absorption and an increase in viscosity until peak viscosity was reached. Subsequent heating caused the starch granules to remain swollen until rupture, releasing starch and reducing viscosity. During cooling, amylose condensation occurred, re-forming hydrogen bonds and increasing viscosity.

The gelatinization temperature marks the point at which the viscosity of the mixture begins to increase, indicating the resistance of starch granules to swelling [37]. This reaction involved the swelling and structural breakdown of rice flour particles, leading to a sudden rise in viscosity. As shown in Table 4, gelatinization temperature and time decreased with decreasing rice flour particle size. This was attributed to larger particles of rice flour presenting a greater physical barrier to heat transfer compared to smaller particles. Consequently, larger particles exhibited a slower hydration rate, requiring more time to develop viscosity during heating. Therefore, rice flour with larger particle sizes tended to have higher gelatinization temperatures and longer gelatinization times [38]. As the particle size of rice flour decreased, its specific surface area increased, providing a larger contact area with water. Simultaneously, mechanical forces increased the content of damaged starch in rice flour, resulting in looser particle surfaces. This made it easier for water molecules to penetrate the rice flour particles, leading to reduced gelatinization temperature and time.

When rice flour particles reached their maximum expansion, achieving equilibrium, the resulting viscosity was known as peak viscosity. It could be indicated that peak viscosity and final viscosity initially increased and then decreased with decreasing particle size. Generally, rice flour with smaller particle sizes tends to exhibit higher gelatinized viscosity [21]. It has been demonstrated that in the preparation of gluten-free foods, the fine powder fraction of rice flour played a crucial role, as its high expansion and gelatinized viscosity were key properties [39]. Gelatinized starch in this fraction bonded together to form the main structural elements of rice flour cakes, and the high gelatinized viscosity aided in maintaining air during heating. After reaching the gelatinization temperature, rice flour particles underwent two processes. One part continuously absorbed water and expanded, while the other part reached its limit and broke down, releasing amylose from the granules and fully extending amylopectin. Consequently, viscosity continued to increase. When the particle size was between 160 and 180 mesh, peak viscosity began to decrease. This decrease was due to the reduction in particle size, increase in damaged starch, and brittleness of the surface of damaged starch particles. These factors resulted in lower resistance to shear forces, weakening the interaction between starch molecules and reducing viscosity. Consequently, together with the RVA curves, rice flour of 140–160 mesh exhibited optimal gelatinization characteristics.

The breakdown value indicates the extent of disintegration of the starch granule structure and the stability of the hot paste during heating. A larger breakdown value suggests a less stable starch structure [17]. The setback value reflects the aging trend of rice flour paste at low temperatures or the stability of a cold paste. The breakdown value increased with decreasing particle size, indicating poor thermal stability of starch during gelatinization (Table 4). This phenomenon was attributed to the loose structure and low strength of starch particles [40]. Additionally, the setback value gradually increased with decreasing particle size. This trend was due to the increased degradation of amylopectin after gelatinization, leading to a reduction in its branch structure. The larger branch structure of amylopectin was not conducive to the starch setback and condensation. Therefore, a decrease in particle size resulted in a higher setback value [41].

### 3.2. Effect of Mesh Sizes on the Properties of Rice Cakes

#### 3.2.1. Texture Properties

In texture profile analysis (TPA) tests, samples undergo two consecutive cycles, during which hardness, elasticity, cohesion, and chewiness are the most common cake parameters that can comprehensively reflect the sample’s ability to resist chewing and significantly impact the eating quality of the cake. Therefore, they were considered the main research parameters. The results indicated that as the particle size of rice flour decreased, the hardness of the cake gradually decreased (Table 5). This could be explained by the rapid gelatinization of starch. Rice flour with smaller particle sizes had a lower gelatinization temperature and began gelatinization earlier, which helped retain effective bubbles, resulting in lower cake hardness and chewiness [42], further confirmed in subsequent correlation analyses. Elasticity and springiness indicated the ability of the cake to return to its original shape and height after the initial compression [43], and both increased with decreasing particle size of rice flour. This was because cakes made from smaller particle sizes of rice flour had higher extensibility [44].

#### 3.2.2. Specific Volume and Porosity

Specific volume is an important indicator for evaluating the quality characteristics of cakes. The larger the specific volume, the better the quality of the cake, which is related to the initial air incorporation and bubble stability of the cake batter [45]. The reduction in particle size significantly improved the specific volume, with cakes made from 120–140 mesh rice flour to 140–160 mesh rice flour showing a more pronounced increase in specific volume (Table 6). The specific volume of the cake depended on the amount of air incorporated during mixing and the amount of gas retained during baking, which was determined by the starch gelatinization characteristics. The texture structure of the cake mainly refers to the compactness of the bread section, which affects the specific volume, hardness, and sensory characteristics of the cake. Cakes with higher porosity are generally considered to have higher baking quality [46]. It can be observed that as the particle size decreased, the porosity and other indices of the cake gradually increased. Cakes with higher porosity had the lowest conductivity, which was consistent with the previous findings [47]. The porosity of cakes made from 160 to 180 mesh rice flour was the highest, indicating a more porous cake structure, which led to a higher degree of softness. Furthermore, observing the texture structure, cakes made from 160 to 180 mesh rice flour had larger pores and a more open internal structure, while cakes made from 100 to 120 mesh rice flour had smaller pores and a relatively dense internal structure. This indicated that the quality of the cakes made from rice flour after fine grinding treatment has been effectively improved.

#### 3.2.3. Color Analysis and Sensory Evaluations

The color parameters of rice flour cakes made from rice flour from different mesh sizes were observed (Table 7). As the particle size of rice flour decreased, the L* and h values of the rice cake surface gradually decreased, indicating a darker color and reduced lightness, while the a* and b* values significantly increased (*p* < 0.05). The increase in a* and b* values was particularly significant between the 120–140 mesh and 140–160 mesh sizes, with a* values increasing from 4.69 to 8.99 and b* values increasing from 33.04 to 37.92. This was due to the increased damage to starch content caused by the reduction in particle size of rice flour, making it more susceptible to degradation by amylases, which produced reducing sugars. Reducing sugars are the substances responsible for the Maillard and caramelization reactions, which are the basis of the browning effect, thereby altering the color of the rice cake. Since the surface is an important site for color reactions, the a* and b* values of the rice cake surface increased significantly [47]. The changes in the core of the rice cake followed a similar pattern to the surface, albeit with slightly lower values. When the particle size of rice flour was in the 140–160 mesh range, the color angle (h value) of the cake changed significantly. This demonstrated that the particle size of rice flour had a significant impact on the color of the surface and core of rice cakes, with a more pronounced effect on the core.

**Figure 5:**
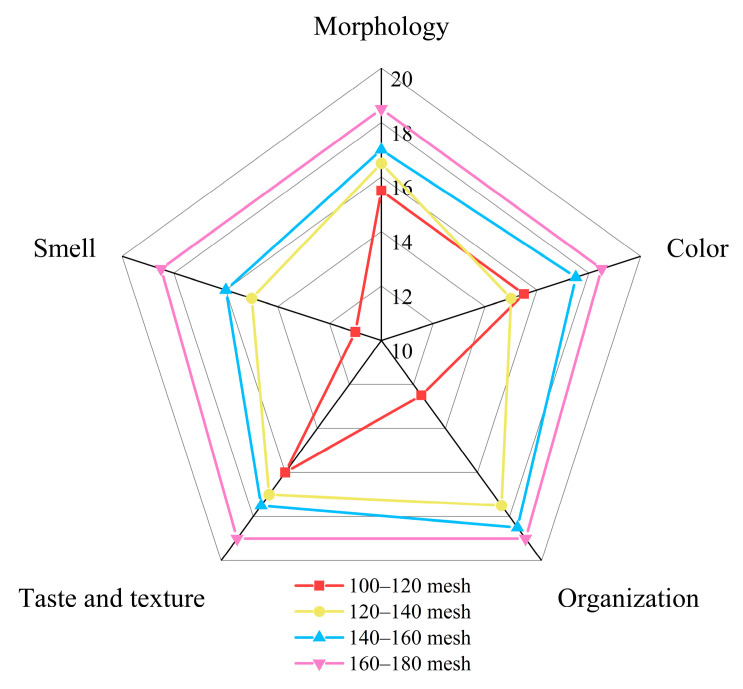
Sensory scores of cakes made from rice flour sieved through different mesh sizes.

The sensory properties of rice cakes made from four different grain sizes of rice flour were evaluated (Figure 5). The results indicated that rice cakes made from 160 to 180 mesh rice flour received the highest sensory scores across all indicators. These cakes were noted for their soft, delicious texture, unique flavor profile, and overall superior sensory evaluation. In contrast, cakes made from 100 to 120 mesh rice flour exhibited an uneven internal structure, coarse texture, flat specific volume, and inferior taste. Finer rice flour produced batter with a lower specific volume and smaller, more uniform bubbles, contributing to a better sensory score [48]. In conclusion, the grain size of rice flour significantly impacted the appearance, shape, color, structure, taste, and mouthfeel of rice cakes, as well as impurities and overall quality.

#### 3.2.4. Correlation Analysis

**Figure 6:**
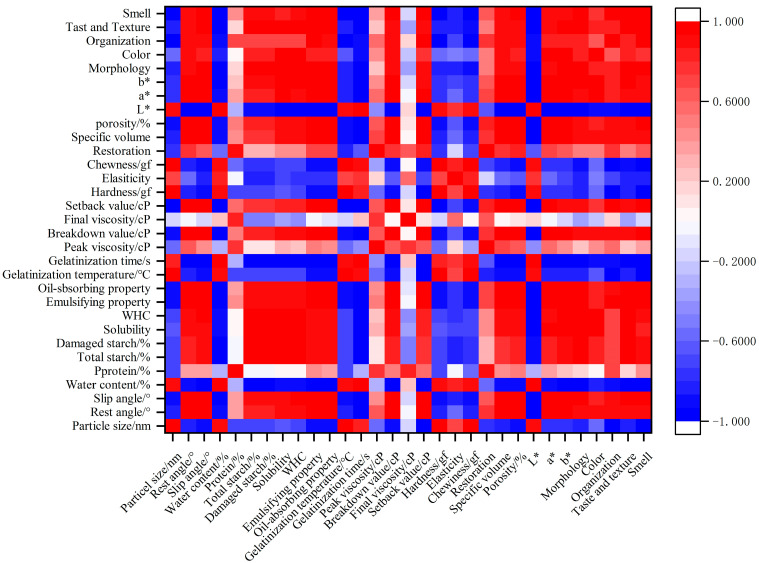
Correlation analysis between different mesh sizes and rice flour cake quality indices.

Through Pearson analysis, the correlation between rice flour particle size and cake quality characteristics was examined (Figure 6). The rice flour particle size exhibited a positive correlation with hardness and gelatinization temperature, a significantly positive correlation with chewiness, a negative correlation with texture, and a significantly negative correlation with setback values.

Moisture content showed a positive correlation with L* value and gelatinization time and a negative correlation with aroma, flavor, appearance, and oil absorption and emulsification. Total starch and damaged starch were significantly positively correlated with flavor, aroma, appearance, WHC, and solubility. The water absorption rate of damaged starch was five times that of normal starch. As the damaged starch content increased, the WHC of rice flour increased, leading to a higher water absorption rate of rice flour. This, in turn, enhanced the WHC and solubility of fine rice flour, thereby affecting the shape and flavor of the rice cake after baking, as well as the Maillard and caramelization reactions.

The rest angle exhibited positive correlations with a* value, specific volume, breakdown value, b* value, porosity, setback value, oil absorption, emulsification, and slip angle, as well as with L* value and gelatinization time. The slip angle showed positive correlations with aroma, oil absorption, and emulsification. Conversely, aroma and taste, appearance, b* value, porosity, breakdown value, setback value, L* value, gelatinization time, and moisture content were significantly negatively correlated with slip angle.

Solubility demonstrated positive correlations with WHC and significantly positive correlations with color and b* value. WHC was significantly positively correlated with flavor, aroma, appearance, b* value, and gelatinization time. Emulsification and oil absorption showed positive correlations with each other and were significantly positively correlated with aroma and flavor, appearance, morphology, b* value, a* value, porosity, specific volume, breakdown value, and setback value. Emulsification was significantly negatively correlated with L* value and gelatinization time, while oil absorption was significantly negatively correlated with L* value and gelatinization time. The correlation analysis indicated that the WHC and solubility of rice flour influenced the shape and flavor of the final rice cake. Additionally, oil absorption and emulsification affected the gelatinization time, breakdown value, and setback value in its gelatinization characteristics. Moreover, oil absorption and emulsification also influenced the shape and flavor of the rice cake, thereby impacting the quality of the final product.

Gelatinization temperature exhibited positive correlations with hardness, chewiness, and texture, and negative correlations with setback value. Gelatinization time showed a positive correlation with L* value and negative correlations with aroma and flavor, flavor, appearance, b* value, a* value, porosity, setback value, and breakdown value. Breakdown value was positively correlated with porosity and specific volume, as well as with b* value, a* value, and setback value, and negatively correlated with L* value. Setback value showed positive correlations with porosity, and with aroma, texture, and specific volume, while being negatively correlated with L* value. Hardness was positively correlated with chewiness and negatively correlated with texture. Chewiness was significantly negatively correlated with aroma and texture.

Specific volume was positively correlated with a* value and porosity, as well as with b* value, and negatively correlated with L* value. Porosity was significantly positively correlated with b* value and a* value and negatively correlated with L* value. L* value was significantly negatively correlated with aroma, flavor, appearance, b* value, and a* value. A* value was significantly positively correlated with color and b* value. B* value was significantly positively correlated with flavor, color, and shape. The color of rice cake was primarily influenced by Maillard and caramelization reactions; hence, the color index was closely related to flavor and aroma. Morphology was positively correlated with flavor and significantly positively correlated with aroma. Additionally, texture was positively correlated with aroma.

## 4. Conclusions

The mesh size of rice flour had an impact on both the characteristics of rice flour and the quality of the rice cake. As the particle size decreased, the basic components, microstructure, and physiochemical properties of fine rice flour were all influenced, leading to significant differences in the rice cake texture and product quality. Rice cakes made from higher mesh number rice flour showed improvements in texture characteristics, specific volume, porosity, and sensory evaluation, scilicet cake made from 160 to 180 mesh rice flour achieved optimal product quality in this study.

Through correlation analysis, several key findings emerged regarding the impact of rice flour characteristics on rice cake quality. Firstly, the resting angle of rice flour influenced its oil absorption and emulsification, which in turn affected its gelatinization characteristics, ultimately improving the final product’s quality. Additionally, oil absorption and emulsification also played a role in shaping the texture and flavor of the rice cake. Secondly, rice flour with smaller particle sizes exhibited a lower gelatinization temperature and initiated gelatinization earlier. This early gelatinization helped retain effective bubbles, leading to lower cake hardness and chewiness. Lastly, WHC and solubility were critical factors that influenced the rice cake’s shape and taste. Consequently, the findings demonstrated that the application of micro-crushing technology significantly enhanced the quality of rice cakes.

## Figures and Tables

**Table 1:** Sensory evaluation criteria of rice flour cake.

Item	Scoring Criteria	Scores
Shape	Neat and full in shape, with a smooth surface and a flat bottom without damage or shrinkage.	15~20
	Relatively neat in shape, with a slight collapse on the top and some minor surface damage.	10~15
	Irregular in shape, uneven in thickness, with a rough surface, and collapse or shrinkage on the top.	<10
Color	The surface is light yellow, and the interior is white, with uniform color, exhibiting the characteristic color features of this variety.	15~20
	Color is relatively uniform	10~15
	Color is not uniform	<10
Texture	Even fermentation, soft and fine texture, good elasticity, fine and non-grainy powder, no sugar lumps, powder lumps, etc., showing a fine and dense honeycomb-like cross-section.	15~20
	Poor fermentation, coarse texture, with larger air holes, and slightly inferior elasticity.	10~15
	No fermentation, no elasticity, with sugar lumps, powder lumps, etc.	<10
Flavor and Taste	Soft and delicious, with a hint of egg aroma, non-sticky, moderate sweetness, with the unique flavor of the rice flour cake, no peculiar flavors, and a light rice aroma.	15~20
	Light egg aroma, slightly hard texture, the unique flavor of rice flour cake is not prominent.	10~15
	Light egg aroma, poor texture, and the unique flavor of rice flour cake is not prominent.	<10
Smell	Pure, authentic, and rich in rice flour cake aroma.	15~20
	The aroma is not strong, with a subtle rice flour cake aroma	10~15
	No cake aroma or presence of off-odors	<10

**Table 2:** Basic nutritional components of rice flour sieved through different mesh sizes (dry base).

Components	100–120 Mesh	120–140 Mesh	140–160 Mesh	160–180 Mesh
Water content (%)	11.25 ± 0.08 ^a^	10.61 ± 0.04 ^b^	10.40 ± 0.11 ^c^	9.80 ± 0.14 ^d^
Protein (%)	7.52 ± 0.04 ^a^	7.63 ± 0.12 ^a^	7.83 ± 0.09 ^a^	7.21 ± 0.13 ^a^
Damaged starch (%)	18.01 ± 0.04 ^a^	19.07 ± 0.11 ^b^	20.08 ± 0.08 ^c^	26.05 ± 0.12 ^d^
Total starch (%)	74.03 ± 1.53 ^a^	73.47 ± 2.63 ^a^	74.37 ± 0.83 ^a^	74.27 ± 1.83 ^a^
D50/μm	125.30 ± 37.11 ^a^	103.30 ± 12.52 ^b^	82.70 ± 11.21 ^c^	71.60 ± 8.50 ^d^
Microscope (×200)	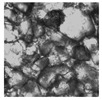	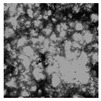	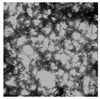	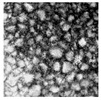

Different letters in each group of data represent statistically significant differences (*p* < 0.05).

**Table 3:** Conductivity (µs/cm) comparisons of rice flour, rice flour paste and rice cake batter sieved through different mesh sizes with different moisture volume fractions.

Materials	Mesh Sizes	Moisture Volume Fraction (%)				
		20	40	60	80	100
Rice flour	100–120 mesh	98.04 ± 3.51 ^d^	60.02 ± 1.36 ^d^	47.19 ± 1.32 ^c^	42.01 ± 0.01 ^d^	36.19 ± 0.83 ^d^
	120–140 mesh	99.04 ± 2.47 ^c^	67.05 ± 1.01 ^c^	54.09 ± 1.45 ^b^	46.06 ± 0.59 ^c^	41.67 ± 0.51 ^c^
	140–160 mesh	102.03 ± 2.01 ^b^	70.06 ± 2.57 ^b^	54.05 ± 0.39 ^b^	45.06 ± 0.14 ^b^	40.66 ± 0.83 ^b^
	160–180 mesh	108.01 ± 3.78 ^a^	71.13 ± 1.54 ^a^	56.08 ± 0.93 ^a^	47.17 ± 0.83 ^a^	43.04 ± 0.38 ^a^
Rice flour paste	100–120 mesh	168.35 ± 4.27 ^a^	133.21 ± 2.29 ^a^	116.75 ± 3.51 ^a^	87.2 ± 1.64 ^a^	72.01 ± 0.11 ^a^
	120–140 mesh	151.24 ± 4.01 ^b^	119.23 ± 1.51 ^b^	89.05 ± 2.82 ^b^	67.24 ± 1.74 ^b^	54.66 ± 0.07 ^b^
	140–160 mesh	143.04 ± 2.76 ^c^	108.09 ± 1.57 ^c^	87.04 ± 1.83 ^c^	63.52 ± 1.83 ^c^	47.09 ± 1.53 ^c^
	160–180 mesh	121.2 ± 1.71 ^d^	91.61 ± 0.35 ^d^	86.23 ± 1.71 ^d^	58.02 ± 0.51 ^d^	43.09 ± 1.71 ^d^
Rice cake batter	100–120 mesh	1540.68 ± 11.35 ^a^	1298.73 ± 7.66 ^a^	1161.53 ± 6.84 ^a^	1023.26 ± 10.43 ^a^	913.81 ± 6.47 ^a^
	120–140 mesh	1494.20 ± 8.44 ^b^	1281.29 ± 9.47 ^a^	1115.17 ± 9.25 ^b^	998.30 ± 8.45 ^b^	880.35 ± 7.79 ^b^
	140–160 mesh	1525.45 ± 12.14 ^a^	1342.04 ± 11.35 ^b^	1144.33 ± 8.73 ^a^	1004.21 ± 7.62 ^c^	905.68 ± 6.88 ^a^
	160–180 mesh	1522.37 ± 13.87 ^a^	1334.21 ± 10.92 ^b^	1163.46 ± 9.46 ^a^	1023.36 ± 11.25 ^a^	924.53 ± 7.57 ^a^

Different letters in each group of data represent statistically significant differences (*p* < 0.05).

**Table 4:** Primary gelatinization factors fine rice flour sieved through different mesh sizes.

Sample	Gelatinization Temperature/ °C	Gelatinization Time/ s	Peak Viscosity/ cP	Breakdown Value/ cP	Final Viscosity/ cP	Setback Value/ cP
100–120 mesh	76.63 ± 0.51 ^a^	6.6 ± 0.15 ^a^	2937 ± 50.61 ^a^	71.8 ± 0.26 ^a^	3254 ± 32.74 ^a^	1089 ± 15.84 ^a^
120–140 mesh	72.61 ± 0.28 ^b^	6.53 ± 0.39 ^b^	3013 ± 48.01 ^b^	82.6 ± 0.41 ^b^	3325 ± 42.12 ^b^	1188 ± 36.73 ^b^
140–160 mesh	71.8 ± 0.64 ^c^	6.47 ± 0.23 ^c^	3343 ± 49.32 ^c^	96.1 ± 0.42 ^c^	3427 ± 39.01 ^c^	1257 ± 26.81 ^c^
160–180 mesh	71.61 ± 0.24 ^d^	6.4 ± 0.57 ^d^	3027 ± 38.21 ^d^	98.8 ± 0.27 ^d^	3166 ± 32.27 ^d^	1273 ± 37.74 ^d^

Different letters in each group of data represent statistically significant differences (*p* < 0.05).

**Table 5:** Texture properties of fine rice cake made from rice flour sieved through different mesh sizes.

Sample	Hardness/gf	Elasticity	Chewiness/gf	Restoration
100–120 mesh	480.11 ± 12.36 ^a^	0.48 ± 0.01 ^a^	297.28 ± 13.21 ^a^	0.33 ± 0.01 ^b^
120–140 mesh	349.77 ± 10.22 ^b^	0.51 ± 0.02 ^c^	217.194 ± 12.03 ^b^	0.35 ± 0.02 ^c^
140–160 mesh	336.44 ± 13.38 ^c^	0.55 ± 0.02 ^b^	219.70 ± 9.42 ^b^	0.38 ± 0.01 ^a^
160–180 mesh	324.91 ± 6.38 ^d^	0.57 ± 0.01 ^d^	195.01 ± 4.01 ^d^	0.38 ± 0.01 ^c^

Different letters in each group of data represent statistically significant differences (*p* < 0.05).

**Table 6:** Specific volume, pore properties, and texture view of cakes made from rice flour sieved through different mesh sizes.

Samples	100–120 Mesh	120–140 Mesh	140–160 Mesh	160–180 Mesh
Rice cake sectional view and black–white analytical view	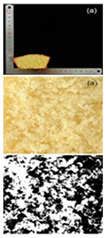	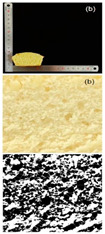	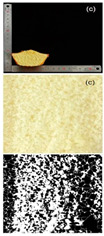	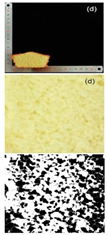
Specific volume (mL/g)	1.38 ± 0.03 ^d^	1.51 ± 0.02 ^c^	1.79 ± 0.01 ^b^	1.81 ± 0.01 ^a^
Porosity (%)	4.82 ± 0.11 ^d^	5.34 ± 0.08 ^c^	5.94 ± 0.02 ^b^	6.03 ± 0.12 ^a^
Pore number	152 ± 2.00 ^d^	191 ± 1.00 ^c^	214 ± 2.00 ^b^	247 ± 1.00 ^a^
Total pore area/um^2^	6558.866 ± 435.86 ^d^	7123.926 ± 456.86 ^c^	8170.297 ± 367.15 ^b^	8593.641 ± 378.74 ^a^
Pore area ratio/%	4.938 ± 0.01 ^d^	5.494 ± 0.01 ^c^	6.139 ± 0.01 ^b^	6.641 ± 0.01 ^a^

Different letters in each group of data represent statistically significant differences (*p* < 0.05).

**Table 7:** Color parameters of rice cake surface and core.

	L*	a*	b*	h (°)
Rice cake surface				
100–120 mesh	78.03 ± 1.41 ^a^	3.32 ± 1.34 ^d^	31.23 ± 0.31 ^d^	83.93 ± 1.01 ^a^
120–140 mesh	74.80 ± 1.72 ^b^	4.69 ± 1.96 ^c^	33.04 ± 0.69 ^c^	81.92 ± 0.56 ^b^
140–160 mesh	72.22 ± 2.31 ^c^	8.99 ± 2.06 ^b^	37.92 ± 0.82 ^b^	76.66 ± 0.32 ^c^
160–180 mesh	69.32 ± 1.36 ^d^	10.08 ± 1.37 ^a^	40.98 ± 0.37 ^a^	76.18 ± 1.78 ^c^
Rice cake core				
100–120 mesh	77.43 ± 0.47 ^a^	0.27 ± 0.01 ^d^	22.77 ± 0.01 ^d^	89.32 ± 1.29 ^a^
120–140 mesh	73.59 ± 0.47 ^b^	0.34 ± 0.01 ^c^	24.31 ± 0.21 ^c^	89.20 ± 0.57 ^a^
140–160 mesh	71.91 ± 0.41 ^c^	0.74 ± 0.01 ^b^	25.53 ± 0.07 ^b^	88.34 ± 2.38 ^b^
160–180 mesh	67.17 ± 0.72 ^d^	2.79 ± 0.01 ^a^	32.31 ± 0.01 ^a^	85.06 ± 2.91 ^c^

Different letters in each group of data represent statistically significant differences (*p* < 0.05).

## Data Availability

The original contributions in this study are included in the article, more inquiries can be directed to the corresponding author upon reasonable request.
